# The Impacts of COVID-19 Restrictions on Quality Adjusted Life Years (QALY): Heterogeneous effects and post-pandemic recovery

**DOI:** 10.1371/journal.pone.0300891

**Published:** 2024-03-28

**Authors:** Raimundo Atal, Paula Bedregal, José A. Carrasco, Felipe González, Rodrigo Harrison, Cecilia Vizcaya

**Affiliations:** 1 Department of Environmental Studies, New York University, New York City, NY, United States of America; 2 Facultad de Medicina, Pontificia Universidad Católica de Chile, Santiago, Chile; 3 Escuela de Negocios, Universidad Adolfo Ibañez, Santiago, Chile; 4 School of Economics and Finance, Queen Mary University of London, London, England; 5 Escuela de Negocios, Universidad Adolfo Ibañez, Santiago, Chile; 6 Department of Pediatric Infectious Diseases and Immunology, School of Medicine, Pontificia Universidad Católica de Chile, Santiago, Chile; OHE: Office of Health Economics, UNITED KINGDOM

## Abstract

**Objectives:**

Estimate the effects of non-pharmacological interventions used to prevent the spread of COVID-19 on the quality of life, measured by Quality Adjusted Life Years (QALYs).

**Methods:**

A survey on 1,506 heads of households from Chile in May of 2022. Respondents were asked basic socioeconomic questions and a version of the EQ-5D-5L questionnaire that was used to calculate the evolution of HRQoLs. Comparisons of means in HRQoLs measures before the pandemic, at the peak of restrictions, and at the moment of the survey were performed.

**Results:**

The average HRQoL of the population before the pandemic was similar to other countries in the region (0.96). At the peak of restrictions (June 2020–August 2021), the average HRQoL decreased to 0.87 (-9%). At the time of survey (May 2022), the average HQRoL was 0.91 (4%). Assuming the recovery trend continued, pre-pandemic HRQoLs would be reached by January 2024. Altogether, the pandemic would have reduced QALYs by 0.2 in average. The effect is larger and the recovery slower among women. Our estimates imply that the restrictions to manage the pandemic came at a cost of 2.4 months of life years for the average (surviving) person, 1.8 months for men and 3.4 for women.

**Conclusions:**

Our results suggest that COVID-19 had worse effects on life quality than previously thought. These effects are more significant among women than among men. Efforts to improve life quality and speed up its recovery could have large positive consequences for the population.

## Introduction

The COVID-19 pandemic resulted in more than 750 million confirmed cases and almost 7 million deaths worldwidec(https://covid19.who.int/). Before the vaccine was available, a widely used strategy to “flatten the curve” consisted of strong non-pharmacological interventions such as lockdowns, quarantines, contact tracing, physical distancing, etc. Some countries employed these interventions in parallel with their immunization strategies once the vaccines were available.

Some estimates suggest that in the early phase of the pandemic, these restrictions averted roughly 530 million infections globally [1]. Additionally, in countries with less stringent policies such as Sweden and the United Kingdom, excess mortality rates were significantly higher [2, 3]. While essential for preventing the spread of the virus, these strategies might have come at important costs to the population. For example, the restrictions led to massive job losses and dramatic lifestyle changes that could have had significant effects on mental health [4, 5].

In many countries, the pandemic exposed weaknesses in social and healthcare systems [6, 7] and confirmed the role of social determinants of health [8]. Factors such as low income, living in crowded homes, relying on public transportation, etc., are all associated with a higher risk of being infected with COVID-19, as well as increased mortality [9, 10]. Socioeconomic variables are also relevant determinants of the potential effects of the non-pharmacological strategies used to prevent the spread. For example, low-income households might have found it harder to work from home and take care of children when living in crowded houses [11]. However, empirical and quantitative evidence about this heterogeneity is scarce, and understanding these impacts can be important for global public health and for the design of strategies to deal with future similar events. In the context of high socioeconomic inequality, such as in Latin America—where this study takes place—exploring these heterogeneous effects becomes very relevant for policymaking [10–12].

Even though the pandemic is officially over, there is evidence of potential long-lasting effects across many dimensions, with many unknowns. For example, among infected people, there are well-documented long-term physical and mental health issues [13]. Additionally, the closing of schools might hinder child development long after the effective COVID-related policies are over [14]. Finally, remote work has been disruptive [15]. All this evidence suggests that some potential impacts of COVID might be felt even after the pandemic is over and all restrictions are lifted.

In this paper, we explore the effects of the non-pharmacological interventions implemented to manage the pandemic on people’s quality of life by studying changes in Quality-Adjusted Life Years (QALYs). We calculate QALY changes associated with the restrictions and compare these changes across different population groups. By examining the trajectory of quality of life at different moments in time, our paper sheds light on the likely recovery of quality of life during and after the pandemic.

## Methods

Quality Adjusted Life Years (QALYs) are often used to estimate the effects of a medical intervention on the overall quality of life of a patient. For example, it is commonly used to evaluate the differences between a transplant and dialysis, two alternative treatments to kidney failure [16]. This calculation is at the basis of cost-effectiveness studies of medical treatments. In this paper, we study changes in QALYs to estimate the impacts of the non-pharmacological interventions to manage the spread of COVID-19 on the quality of life in Chile, a middle income country that imposed severe movement restrictions on the population as a central component of its strategy to prevent the spread of virus. We surveyed 1,506 heads of households by phone in May 2022. The survey included basic socioeconomic and demographic questions as well as a version of the EQ-5D-5L questionnaire, a standardized, and widely used instrument to estimate QALYs. Throughout the study we had no access to information that could identify individual participants. Permission to conduct the study was granted by the ethics committee from the Adolfo Ibañez University. Individual verbal consent was obtained and the process was documented and witnessed by the surveyor.

Socioeconomic and demographic questions were based on the *“CASEN en Pandemia”* (CASEN) survey made by the Chilean government in 2020 to monitor the impacts of the pandemic on employment and income. This part of the survey collected data on age, education, household size, changes in employment and income during the pandemic, as well as closeness to the disease, etc. Descriptive statistics are presented in Table 1. Our sample survey is comparable across indicators to national averages as measured by the CASEN database.

**Table 1:** Descriptive statistics†.

	Avg.	St. dev.	Min	Max	CASEN 2020 Average
	(1)	(2)	(3)	(4)	(5)
Age	49.7	13.3	20	89	47.1
Indicator female	0.44	0.50	0	1	0.56
Years of education	12.8	3.7	0	20	11.9
Indicator immigrant	0.13	0.33	0	1	0.07
Individuals	1,506				138,346

^†^Columns 1–4 show descriptive statistics for the main variables used in our analysis. For comparison purposes, we include in column 5 the averages for similar variables in the 2020 CASEN survey.

The EQ-5D-5L questionnaire is a standardized survey that is widely used to calculate QALYs. There, patients are asked to evaluate their health in terms of five dimensions (mobility, self-care, usual activities, pain and discomfort and anxiety or depression) and five levels (from no problems, to lots of problems). With the answers and estimates of “value weights” for each health state, one calculates the *Health Related Quality of Life Index* (HRQoL), a number that goes from 0 to 1, where 0 and 1 represent death and perfect health, respectively. With this, the Quality Adjusted Life Years for an individual are calculated as the product of the time spent in a particular level of the HRQoL index, and its level. For example if a person lives 2 years with a HRQoL of 0.5, this results in 1 Quality Adjusted Life Year. In our version of the EQ-5D-5L survey, respondents were asked to situate themselves in three moments: “*before the pandemic*” (March 2020), “*during the moment of peak restrictions*” (June 2020–August 2021), and “*today*” (May 2022, when most restrictions had been lifted). With this, we calculated the HRQoL index at each point in time. Since there are no estimates of the value weights for the EQ-5D-5L survey in Chile, we used available estimates for Uruguay, a country with similar GDP per-capita. However, we show the robustness of our results in the supporting information section (Table 2) using a crosswalk mapping from the EQ-5D-3L value set from Chile. We use the value set from Uruguay for our main exercise because the EQ-5D-5L is superior to EQ-5D-3L and to crosswalk values in terms of its sensitivity and precision in health status measurement; it provides more precise utility measurements at individual and group levels [17, 18].

**Table 2:** Robustness of results.

Number in paper	EQ-5D-5L (Uruguay)	EQ-5D-3L (Chile)	(1) + deaths
	(1)	(2)	(3)
HRQoL starting point	0.96	0.91	0.96
HRQoL decrease at peak pandemic	0.87	0.77	0.87
HRQoL date of the survey	0.91	0.81	0.91
QALY losses by date of survey	79%	79%	79%
Pandemic reduction of QALYs	0.20	0.35	0.26
Months lost avg. surviving person	2.4	4.2	3.1^a^
Men	1.8	3.4	2.3
Women	3.4	5.5	4.4

**Notes**: EQ-5D-3L values from [39]. Column 3 is calculated by assuming a HRQoL profile for individuals who died at the peak of the pandemic (0.31% of adult population), those who died between the peak and the time of the survey (0.07%), and those who died after the survey date (0.06%).

^*a*^This number is calculated assuming that dead people had 240 months to live at pre-pandemic health; if we use 120 months the number is 2.7 months and 2.5 months if we use 60 months.

In order to estimate the impacts on QALYs, we need to recover the trajectory of HRQoLs over time from the three point estimates discussed above. We do so using data on infections and the monthly share of municipalities that were under a lockdown in Chile during the pandemic. These are illustrated in our supporting information section. We assume that HRQoLs started declining linearly in March 2021, when the first case of COVID-19 was detected, until reaching the level in the period of *“peak restrictions”*. We assume the latter to span between the peak of infections of the first wave (June 2020) until the moment when all lockdowns were lifted (August 2021). Then, we assume HRQoLs to recover linearly as well, crossing the value of HRQoLs at time of the survey (May 2022), until reaching their pre-pandemic levels.

## Results

### HRQoL and QALY losses

**Fig 1:**
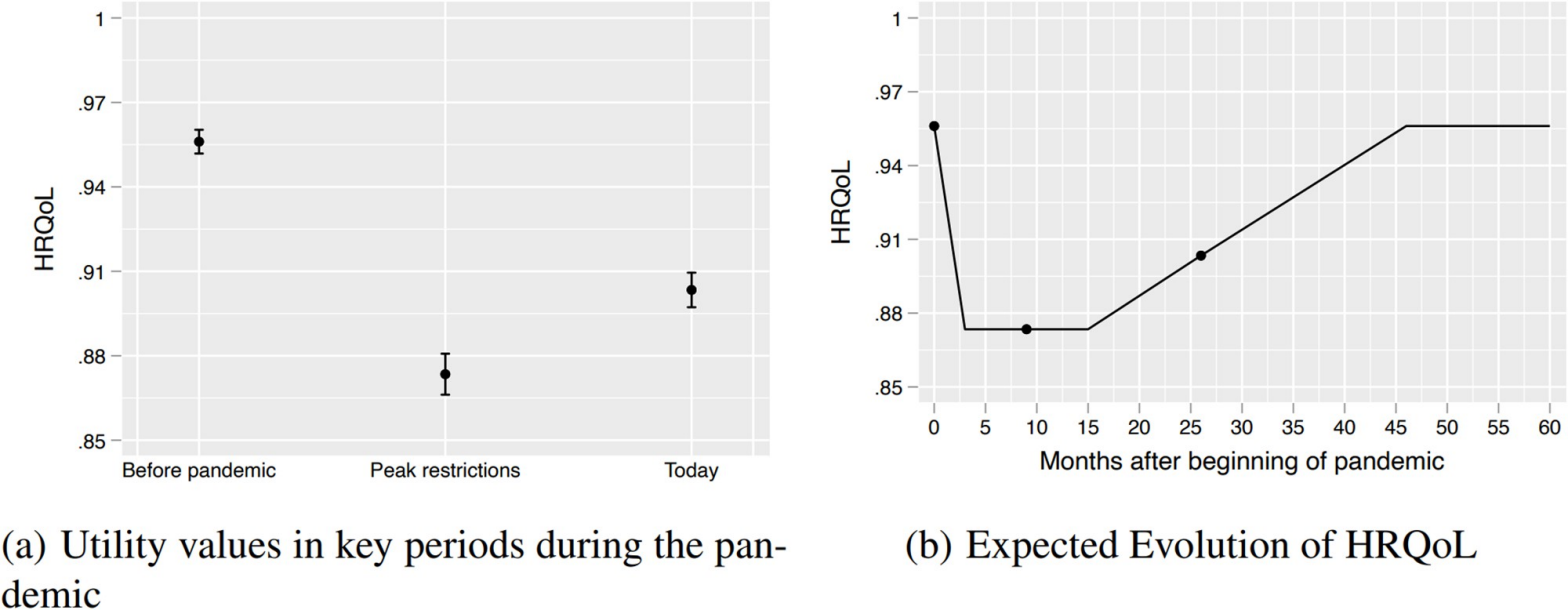
The Effects of COVID-19 on QALY. At left, we show the average (black circles) and 95 percent confidence interval (vertical capped lines) for HRQoL “before the pandemic”, at “peak restrictions” and “today”, which is when the survey took place. At right, we show the expected evolution of HRQoL; pre-pandemic levels should be reached 46 month after the beginning of the pandemic.

Panel (a) in Fig 1 shows the average HRQoL before the pandemic, at the peak of restrictions, and at time of survey, when most restrictions (e.g. lockdowns) were lifted. Before the pandemic, HRQoLs have an average level of 0.956 with a 95% confidence interval (CI) from 0.951 to 0.960. A similar value (0.954) to the one found for the Uruguayan population [19]. The survey reveals that, at the peak of restrictions, HRQoLs fell to 0.873, 95% CI 0.866–0.873. In contrast, by the time of the survey (May 2022), when most restrictions were lifted, average HRQoLs had partially recovered to 0.903 (CI 0.910–0.897) but remained lower than before the COVID-19 outbreak in Chile.

In order to estimate QALY changes we assume a trajectory of HRQoLs during the pandemic as discussed in the methods section. The solid line in panel (b) of Fig 1 shows this simulated trajectory, while the dots correspond to the data points derived from the survey. With this trajectory, we estimate that HRQoL values will return to pre-pandemic levels by January 2024, i.e. 46 months after the first COVID-19 case in Chile. The area below pre-pandemic HRQoL during the 46 months period represent losses in life quality associated to the pandemic and the corresponding policies that were triggered as a consequence. Our results suggest that after return to pre-pandemic levels in HRQoL, individuals will have lost an average of 0.20 of QALYs, or 2.4 months. By the time of survey in May 2022, individuals had lost, on average, 0.16 QALYs, or 1.9 months. This is, we calculate that 79% of the QALY losses had already been realized by the time of the survey.

### QALY losses and recovery by gender

**Fig 2:**
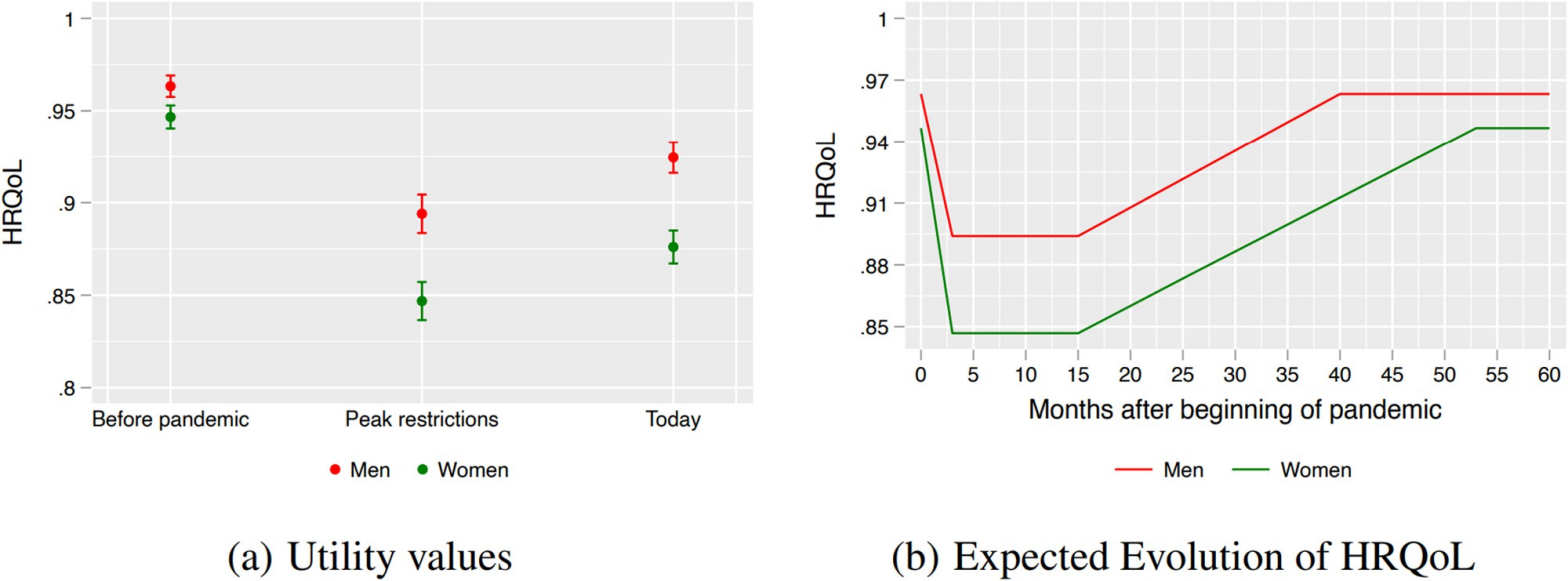
The Effects of COVID-19 pandemic on QALY—By gender. At left we present the average (black circles) and 95 percent confidence interval (vertical capped lines) for HRQoL, our measure of QALY. At right, we show that men should reach pre-pandemic levels after 40 months, while women after 53 months.

Panel (a) in Fig 2 shows the estimated HRQoL values for men and women. Women had, on average, a statistically significant lower quality of life before the pandemic (0.947, CI 0.940–0.953) than men (0.963, CI 0.957–0.969). This estimated gender gap in HRQoL before the pandemic is similar to the one found in Uruguay, i.e. 0.963 versus 0.947 [19]. At the peak of restrictions, the HRQoL index decreased to 0.894 (CI 0.884–0.904) for men and to 0.847 (CI 0.837–0.857) for women, a difference that is statistically significant (*p*-value<0.01).

Given the documented recovery at the time of the survey, we project that men return to pre-pandemic levels more rapidly than women. In particular, we estimate that men will reach pre-pandemic HRQoL levels after 40 months (July 2023) of the first COVID-19 infection. In contrast, women will only reach pre-pandemic HQRoL after 53 months, i.e. in August 2024. This is, it will take more than one additional year for women to return to pre-pandemic quality of life than men. As a consequence, we estimate that by late 2024 men would have lost 0.15 QALYs (1.8 months), while women would have lost 0.28 QALYs (3.4 months).

### QALY losses and recovery by infection

**Fig 3:**
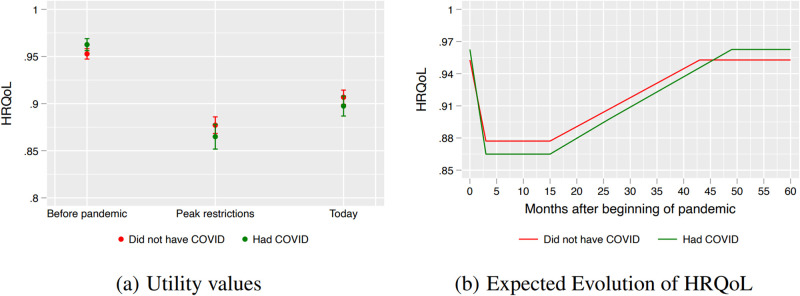
The effects of COVID-19 pandemic on QALY—By COVID infection. At left we present the average (black circles) and 95 percent confidence interval (vertical capped lines) for HRQoL, our measure of QALY. At right, we show trajectories for those that did and those that did not have COVID.

One could expect that decreases in quality of life was stronger for people who were infected with COVID-19. The survey reveals that the HRQoL trajectories and QALY losses are similar across individuals who had been infected by May 2022 and the rest of the population. The measure of COVID-19 infection is based on the survey and it is self-reported by respondents. Panel (a) in Fig 3 shows the average HRQoL across the two groups at each point in time. Individuals previously infected and the rest had similar HRQoL before the COVID-19 pandemic, 0.963 (CI 0.956–0.969) and 0.953 (CI 0.947–0.958) respectively, and there are no statistically significant differences between HRQoLs during the length of the pandemic. Taking the average values as center points for the recovery extrapolations, we estimate that those infected will return to pre-pandemic levels after 49 months (April 2024), a bit longer than the 43 months (October 2023) to full recovery that will take to the rest of the population.

### QALY losses and recovery by age

**Fig 4:**
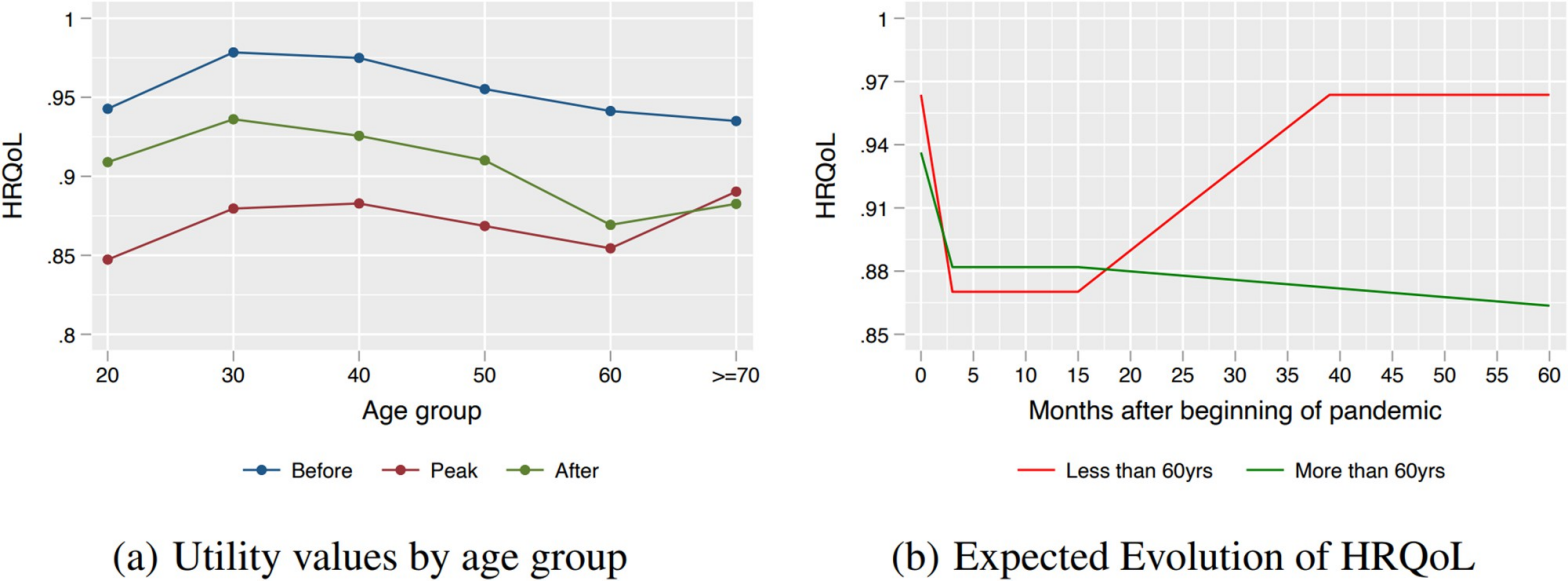
The effects of COVID-19 pandemic on QALY—By age. Panel (a) shows the HRQoL values for different age groups. All individuals suffered a decrease in HRQoL during the pandemic. However, the partial recovery from the peak of restrictions to the time of the survey was smaller among older individuals. Panel (b) shows the QALY trajectory for two age groups. There is a persistent decrease in QALY among the elderly.

The recovery from the peak of restrictions to the time of the survey appears to be more pronounced among younger individuals. Panel (a) in Fig 4 shows the HRQoL values for different age groups. All individuals suffered a decrease in HRQoL during the pandemic. However, the partial recovery from the peak of restrictions to the time of the survey was smaller among older individuals (more than 60 years old). In particular, the recovery was 65% in the 20–30 age bracket, 57% in the 30–40 bracket, 46% in the 40–50 bracket, and 48% in the 50–60 bracket. In contrast, the recovery was only 17% among those 60–70 years old, and -17% among individuals older than 70 years old. Panel (b) in the same figure displays the estimated QALY trajectory for individuals older than 60 years old and the rest. The figure shows that while the youngest recover before the 40th month, the elderly continue to exhibit a decrease in QALY. The persistent decrease after restrictions is a consequence of the relatively low QALY in May 2022 (0.877), which is lower than at the time of the peak of restrictions (0.882).

## Discussion

To the best of our knowledge, this is one of just a few papers to study changes in perceived health using the QALY composite to measure the costs associated with imposed restrictions to manage the COVID-19 pandemic. It is certainly the first to do so for a Latin American country.

We highlight a few contributions. First, our paper provides relevant evidence on the heterogeneous impacts across population groups. In addition, we perform our investigation in Latin America, where restrictions were particularly strict and where a context of high inequality highlights the importance of studying the heterogeneous effects. Economic evaluations of the cost-effectiveness of public health measures to deal with the pandemic have concentrated in high and middle income countries and have overlooked distributional concerns [20, 21]. This makes our contributions particularly relevant, both from an academic perspective as well as an input for policy-making.

We document a greater impact of these restrictions on women. Consistent with previous research, we find that men report higher quality of life than women before the pandemic [22, 23]. Our findings suggest that, on top of that, women experienced stronger reductions in quality of life and a slower recovery process once the restrictions were lifted. These unequal impacts across men and women is consistent with documented inequalities in the experience between men and women during the pandemic. Women were the subjects of increased domestic violence, represent a higher share of essential workers, and disproportionately take care of children, the elderly and the sick [24, 25]. Our results are an important piece of quantitative evidence of increased gender inequality as a result of the policies to manage the pandemic.

We also find that QALY losses are greater among the older population. HRQoLs decreased in all age groups at the peak of the restrictions, with a partial recovery that has been worse in the 70 and older age group. There are a few plausible explanations for this result. First, studies have shown that, in general, older people experience stronger reductions in QALYs related to chronic illnesses [22, 26]. Second, in Chile, during the pandemic, older adults were subject to longer quarantines. This might have affected more not only their physical but also their mental health [27]. Third, we know that in Chile, there was a sharp reduction in the supply of non-COVID related health care services. For example, [28] documents a 40% drop in new cases entering cardiovascular programs in the public health-care system as well as an increase in deaths from cardiovascular diseases [29]. This reduced supply appears to have affected more the older population.

There are two main differences between our results and the ones by [30], who find reductions of HRQoLs in Sweden as a result of the COVID-19 outbreak and imposed restrictions. First, they do not find differences in HRQoLs previous to the pandemic between men and women. In addition, they find that quality of life decreased more among the working-age population. These differences might come from methodological differences as well as context. In particular, they rely mainly on the Visual Analog Scale (VAS) through questionnaires at different times during the pandemic. Instead, we rely on the retrospective EQ-5D-5L measurements and utility weights for health states. Second, we study Chile, a developing country where gender inequalities might be stronger and where the different social welfare systems and the different specific aids given by the state might be behind the differential impacts by age group.

A few limitations signal potential avenues for future research. First, since there are no estimates of utility weights for the Chilean population for the EQ-5D-5L questionnaire, we use the Uruguayan weights, under the assumption that these are similar. Since the valuation of health conditions can be discretionary according to social context [31], it would be valuable to obtain utility weights estimates for EQ-5D-5L from Chile. Despite the fact that we show the robustness of our results using a crosswalk mapping from the EQ-5D-3L value set from Chile (see our Supporting information section), this would be beneficial to other studies that aim at estimating changes in QALYs of other policies or health treatments in Chile.

Regarding the sample under study, we limit our attention to heads of households. This decision was made to have a sufficient sample size given the limited resources. It is not obvious whether this is likely to be a underestimation or overestimation of the impacts of the general population, or other subgroups. It would be then beneficial to perform similar studies with other subgroups as a focus, or a larger sample size than can be representative of the overall population. In the supporting information section (Table 2) we account for the fact that we work with a selected sample that only covers those who survived COVID-19. For, in principle, there might be a bias that arises by not interviewing those who died from COVID-19. However, since in Chile less than 0,5% of adult population died due to COVID-19, our results are robust to this kind of adjustments.

A natural concern is the potential for recall bias in a retrospective survey. Recall bias might lead respondents to overestimate the detrimental effect of a stressful experience [32, 33]. While present in our study, the literature suggests that in the context of HRQoL studies, and the EQ-5D in particular, recall bias is limited, specially when evaluating impacts at the group level rather than individual cases [34–36].

Regarding the use of EQ-5D-5L to measure health-related quality of life, despite being limited in terms of the aspects it assesses, current evidence shows that it is sufficiently robust to detect changes of epidemiological and global clinical significance, making it a frequently used instrument in population surveys [37]. For clinical studies or studies that seek to evaluate the impact of policies and programs, it is suggested to use this instrument and incorporate other instruments that delve deeper into those health domains of interest, for example: chronic pain, depressive symptoms, anxiety, metabolic measurements, among others. Our study can be seen as a first approximation to reveal patterns of impacts of these policies that can be studied deeper in future research.

## Supporting information

S1 Fig The COVID pandemic in Chile: Own construction using administrative data from the Ministry of Health. Vertical gray lines indicate the peak of infections in panel (a) and vertical red lines indicate the period with lockdowns in panel (b). The union of both periods constitutes the time frame that we call “*during the peak of restrictions*,” i.e. June 2020 to August 2021.
(TIFF)

S2 Fig Main results using crosswalk between EQ-3D-5L and EQ-3D-3L for Chile. We use administrative data from the Ministry of Health from Chile, and the crosswalk method from [38].
(TIFF)

S3 Fig Main results including COVID-19 deaths. The adult population in Chile in 2020 was approximately 14.3 million people. The group of survivors of the pandemic are 99.56% of the adult population, 0.31% of the adult population died by the peak of the pandemic (before August 2021), 0.07% between the peak of the pandemic and the survey date (May 2022), and 0.06% after the survey date (after May 2022). Profiles for utility values in panel (a) are assumed based on the distribution of pre-pandemic health and selecting individuals with bad health as those who died (below 5th percentile of the QALY distribution).
(TIFF)
