# Supplementary figures and images for: The Impacts of COVID-19 Restrictions on Quality Adjusted Life Years (QALY): Heterogeneous effects and post-pandemic recovery
Source: PLoS One. 2024 Mar 28;19(3):e0300891. doi: 10.1371/journal.pone.0300891 (PMC10977738; doi:10.1371/journal.pone.0300891)

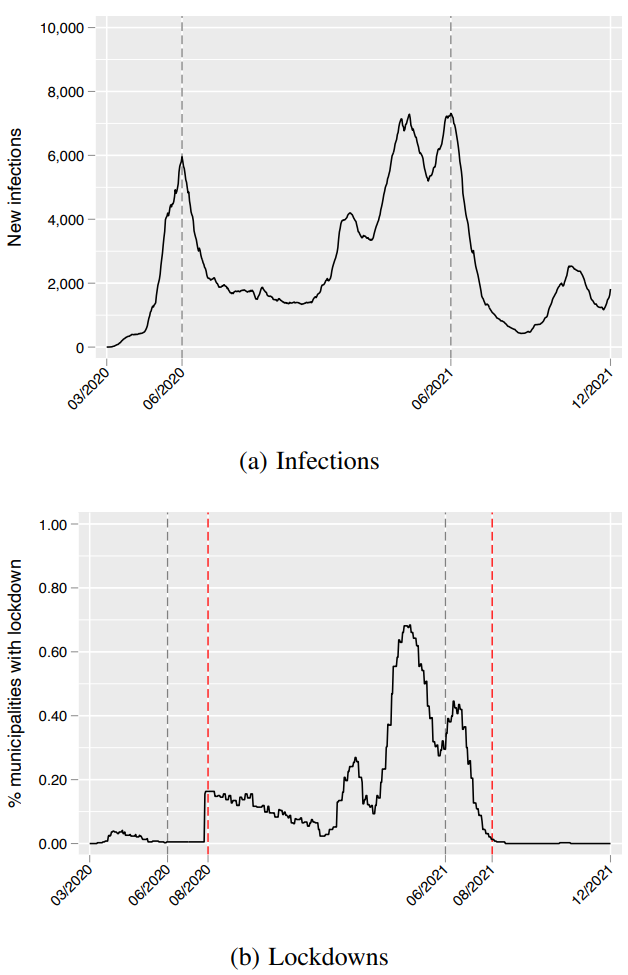

Supplement: S1 Fig — Vertical gray lines indicate the peak of infections in panel (a) and vertical red lines indicate the period with lockdowns in panel (b). The union of both periods constitutes the time frame that we call “during the peak of restrictions,” i.e. June 2020 to August 2021. (TIFF) [file pone.0300891.s001.tiff]

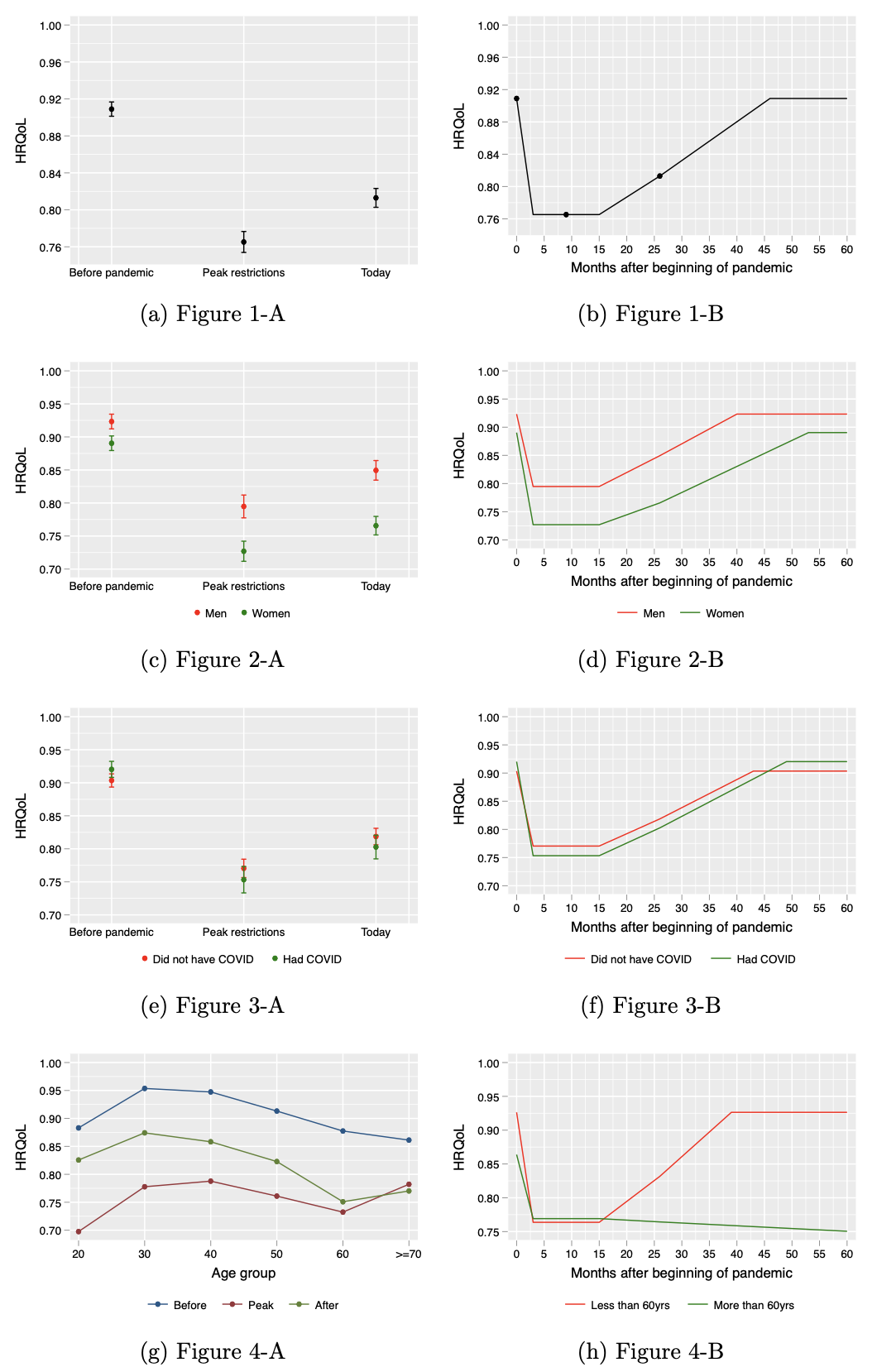

Supplement: S2 Fig — We use administrative data from the Ministry of Health from Chile, and the crosswalk method from [38]. (TIFF) [file pone.0300891.s002.tiff]

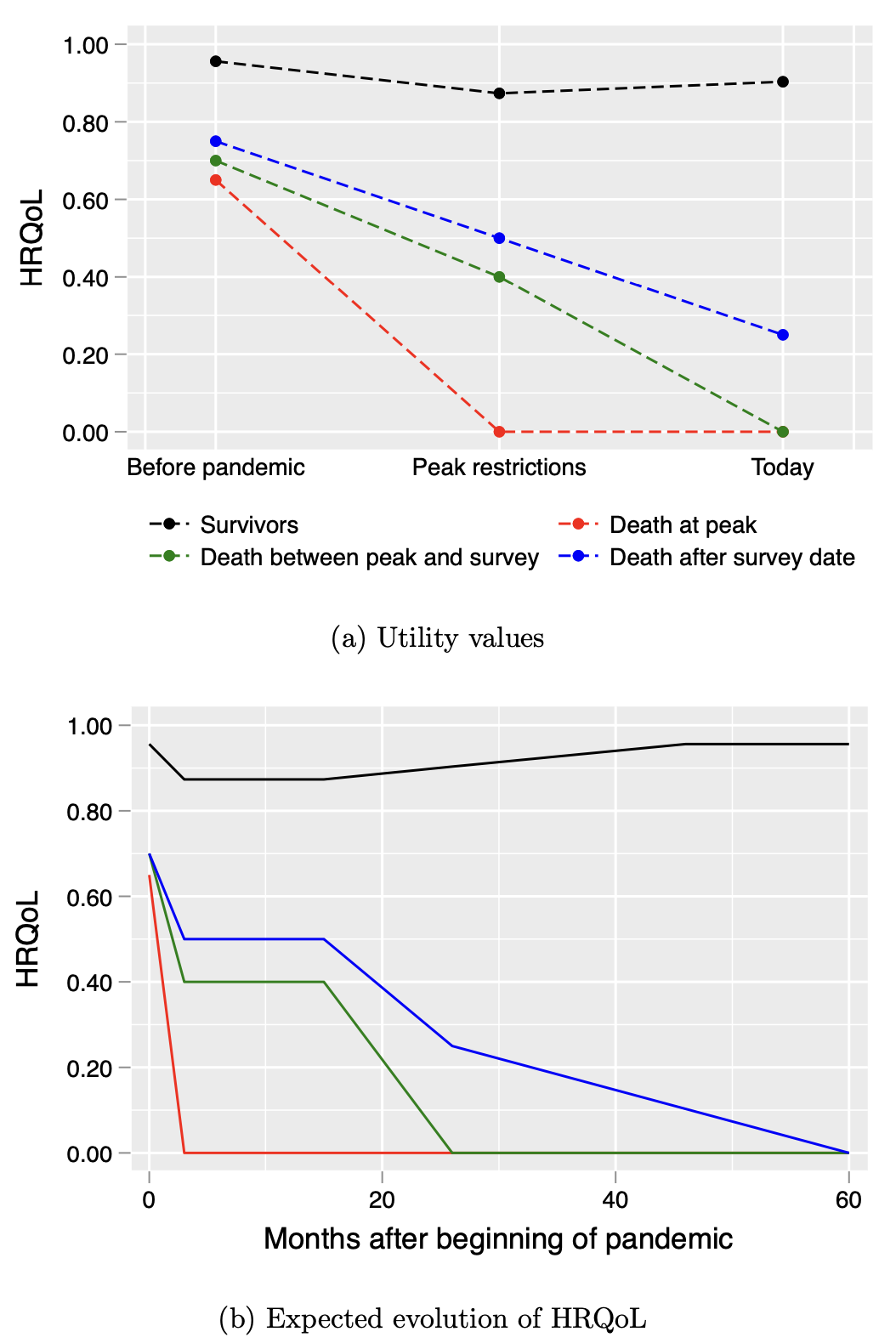

Supplement: S3 Fig — The adult population in Chile in 2020 was approximately 14.3 million people. The group of survivors of the pandemic are 99.56% of the adult population, 0.31% of the adult population died by the peak of the pandemic (before August 2021), 0.07% between the peak of the pandemic and the survey date (May 2022), and 0.06% after the survey date (after May 2022). Profiles for utility values in panel (a) are assumed based on the distribution of pre-pandemic health and selecting individuals with bad health as those who died (below 5th percentile of the QALY distribution). (TIFF) [file pone.0300891.s003.tiff]
